# Long-COVID and postural orthostatic tachycardia syndrome: a preliminary comparison of neuropsychological performance

**DOI:** 10.1007/s10286-025-01106-y

**Published:** 2025-01-22

**Authors:** Aitana Ruiz de Lazcano, Paula Pérez-Núñez, Mercè Pallarès-Sastre, Maddalen García-Sanchoyerto, Irune García, Imanol Amayra

**Affiliations:** Neuro-E-Motion Research Team, Department of Psychology, Faculty of Health Sciences, University of Deusto, 48007 Bilbao, Spain

**Keywords:** SARS-CoV-2, POTS, Dysautonomia, Cognitive assessment, Psychological symptoms

## Abstract

**Purpose:**

The aim of the study is to analyze and compare the cognitive profile between 59 patients with long-COVID [LC; 30 of them with and 29 without a positive coronavirus disease 2019 (COVID-19) confirmatory test] and 31 patients with postural orthostatic tachycardia syndrome (POTS) and a matched group of 39 healthy control participants.

**Methods:**

Participants were examined on a battery of neuropsychological tests, including verbal memory, visuospatial abilities, attention, processing speed, verbal fluency, working memory, and visual memory. Anxious–depressive symptomatology was also analyzed and then controlled for possible influence on cognitive performance.

**Results:**

Patients with LC and POTS showed significantly lower performance compared with healthy peers. Differences on anxious and depressive symptoms were also found between the clinical and control groups, resulting in LC without a positive confirmatory test group exhibiting the highest rates of anxious symptoms. After controlling the effects of anxious–depressive symptomatology, the differences were eliminated for some of the cognitive variables, but additional differences were found between patients with LC and POTS after post hoc analysis.

**Conclusions:**

Findings from the present study contribute toward the reinforcement of the evidence on cognitive alterations associated with LC and POTS. Anxious–depressive symptomatology has to be considered in both clinical groups since it could be affecting cognitive performance.

## Introduction

After surpassing the highest point of the coronavirus disease 2019 (COVID-19) pandemic, multiple post-COVID conditions are starting to be reported by patients with long-COVID (LC) [1–3]. Due to the negative impact of COVID-19 on the autonomic nervous system, one of the most reported sequelae is autonomic disorders [4, 5]. Postural orthostatic tachycardia syndrome (POTS) is the most common type of chronic autonomic disorder [2, 6–9] causing severe disability [10–14]. Due to the increasing number of cases, POTS is gaining recognition in patients with COVID-19 in the postinfectious phase [15]. There is growing concern that, in addition to LC, we may also face a rise in POTS cases [16].

The complex interplay between viral infections and the development of POTS is evident [16, 17]. While various stressors (including physical trauma, hormonal changes, and surgery) [17, 18] or genetic predisposition [19] can contribute to POTS, viral illnesses, particularly those leading to persistent symptoms, appear to be a common pathway to the development of this condition [16, 20–22]. In this way, both LC and POTS are complex, multisystem conditions [23, 24] with a significant symptom overlap [25]. These two groups of patients have to face symptoms that affect the cardiovascular, pulmonary, gastrointestinal, and nervous systems [26, 27].

A preponderance is observed in young and middle-aged women, although they can occur in any sex and age [28, 29]. Given the higher prevalence in women compared with men in both clinical populations, studies have been conducted to identify potential sex differences in symptomatology and functional impact. Findings from published studies suggest that women with a LC diagnosis are more likely to experience a greater number of persistent symptoms than men with LC, regardless of the severity of their initial illness or whether they were hospitalized or not [30, 31]. Women with LC also seem to experience a more significant functional decline when compared with men with LC [32]. Although sex differences have not been extensively studied in the POTS population yet, similar results have been obtained regarding symptomatology. For example, a recent study [33] using self-reported measures revealed a higher number of symptoms among women with POTS compared with men with the same diagnosis. While both genders described the same top ten most frequently reported symptoms, there were notable differences in symptom profiles [33]. Specifically, women with POTS experienced a higher prevalence of symptoms related to muscle pain, while men with POTS reported more sleep disturbances [33]. Further research is needed to identify more potential differences in symptomatology and functional impact between women and men of these two clinical populations.

Psychological similarities can also be identified between LC and POTS, such as higher levels of anxiety and depression [34–40] or lower levels of quality of life [10, 35, 41–46] compared with the general population.

Furthermore, both clinical groups demonstrate shared cognitive impairments. Concerning the LC population, research has allowed identifying the following most common cognitive symptoms in these patients: (a) difficulties in concentrating [47]; (b) memory impairment (including working memory, verbal memory, and visual memory) [48–55]; (c) attention deficits [48–50, 53, 54, 56]; (d) executive dysfunction (specifically on cognitive flexibility, planification, and information processing speed) [49–53, 55, 57]; (e) deficits in visuospatial processing [50, 55]; and finally, (f) issues related to language such as anomia or verbal fluency difficulties [48, 51].

In relation to the patients with POTS, studies have highlighted difficulties in areas such as attention [35, 58, 59], processing speed [35, 58], working memory [60–62], and executive functioning [58, 59]. However, the cognitive profile of patients with POTS is not only an under-researched area, but it is also difficult to reach a conclusion and obtain an accurate cognitive profile in this group, because the results vary significantly among published studies. These differences are due to the neuropsychological tests applied in each of them, as well as to the postural position in which the evaluation was performed, comorbidities, or the time the symptoms have been present [35, 39, 40, 58, 59, 63–65]. Despite these cognitive similarities among both groups, it is not clear whether it is attributable to organic or other factors.

Regarding studies on brain changes in patients with LC, a small amount is available. Research conducted discovered variations in grey matter (GM) volume [66], particularly in the hippocampus [67, 68]. Hypoconnectivity changes in orbitofrontal and parahippocampal areas have also been revealed [66, 69]. These alterations in the parahippocampal areas have been related to the cognitive performance of patients with LC, particularly in attention [66], processing speed [66], and memory [66, 69, 70] tasks. In relation to POTS, there is also limited literature existing about structural brain changes in this population. To date, no studies have been conducted to assess the relationship between these structural changes and the cognitive dysfunction experienced by these patients. However, a study conducted with people with a POTS diagnosis detected structural brain changes in the bilateral regions of the putamen and the cingulate gyrus [71], two areas that seem to be responsible for attentional processes [72, 73].

Although POTS is very common among patients with a LC diagnosis, and despite all the findings mentioned before, there is still insufficient evidence to analyze and compare the neuropsychological profile associated with LC and POTS from a global perspective. Therefore, the main objective of this research is to study and compare the cognitive profile of patients with LC and POTS (analyzing their performance on different cognitive domains including verbal memory, verbal fluency, attention, working memory, visual memory, and spatial cognition) and identify the particularities about each of the clinical groups and see to what extent cognitive performance is related to emotional factors (anxious–depressive symptomatology).

## Methods

### Participants

A convenience sampling method was carried out. There was a total of 129 participants in this sample, divided into a control group and three clinical groups: LC with positive test group (LC^+^) (*n* = 30), LC without positive test group (LC^−^) (*n* = 29) and POTS group (*n* = 31), and a control group (*n* = 39). The LC group was divided into two subgroups: (a) the LC^+^ subgroup that comprised patients who were serologically positive for the severe acute respiratory syndrome coronavirus 2 (SARS-CoV-2) virus after experiencing the first symptoms and (b) the LC^−^ subgroup that included patients who experienced symptoms but did not have a confirmatory test, since there was limited access to serological testing in the first 3 months of the pandemic. This distinction was made to analyze possible differences between the subgroups.

Participants were contacted through the following Spanish associations: Long Covid Euskal Herria Elkartea, Long Covid ACTS (Autonomous Communities Together Spain), and Spanish Association of POTS and other Dysautonomias—Asociación Española de POTS y otras Disautonomías (AEPOD). These participants were recruited between 2020 and 2023, through social media and the associations that have already been mentioned. A total of 39 participants voluntarily signed up from outside the clinical groups made up the group of healthy control participants. The sociodemographic data is presented in Table 1.

**Table 1:** Sociodemographic data of the sample

Variable	LC^+^ group (*n* = 30) *M* (SD)/*n* (%)	LC^−^ group (*n* = 29) *M* (SD)/*n* (%)	POTS group (*n* = 31) *M* (SD)/*n* (%)	Control group (*n* = 39) *M *(SD)/*n* (%)
Sex				
Female	27 (90%)	23 (79.3%)	30 (96.8%)	35 (89.7%)
Male	3 (10%)	6 (20.7%)	1 (3.2%)	4 (10.3%)
Age (years)	45.13 (8.18)	46.76 (6.59%)	42.19 (11.37)	44.59 (8.45)
Educational level	15.67 (2.85)	15.48 (5.35)	15.34 (3.53)	15.33 (2.86)

*LC*^+^ long-COVID with a positive test group, *LC*^*-*^ long-COVID without a positive test group, *n* number of participants, *M* mean, *SD* standard deviation

Regardless of the group to which they belong (clinical or control group), all participants must meet this inclusion criteria: (a) be of legal age, (b) sign the informed consent form before the assessment, and (c) speak Spanish as one of their main languages. The exclusion criteria included: (a) younger than 18 years, (b) presence of any additional diagnoses or sensory deficiencies that would prevent the tests from being applied, (c) drug and/or alcohol abuse, (d) having communication difficulties that may impede and hinder the correct completion of the neuropsychological tests, (e) presence of any additional diagnoses or sensory deficiencies that would prevent the tests from being applied, (f) being illiterate, and (g) diagnosis of any severe or serious mental illness.

Further, in the case of the three clinical groups, the following inclusion criteria were established: (a) for the LC^+^ group, having been infected with COVID-19 confirmed by a positive test and having a LC diagnosis; (b) for the LC^−^ group, having experienced COVID-19 symptoms but not undergoing serological screening for COVID-19 and having a LC diagnosis; and (c) for the POTS group, having a POTS diagnosis before the COVID-19 pandemic. For the control group, the inclusion criteria were: (a) not having been infected by COVID-19 and (b) not having a diagnosis of LC or POTS.

LC participants (from both LC^+^ and LC^−^ groups), received their diagnosis through telemedicine. The reason for this approach (online diagnosis) was the restrictions imposed by various medical centers and units in Spain at the time of data collection. Due to the COVID-19 pandemic, diagnostic visits were prohibited, preventing in-person evaluations during that period. Regarding participants in the POTS clinical group, all of them received a diagnosis from specialized Spanish autonomic dysfunction units or from neurologists or cardiologists from different regions of Spain. Diagnoses were confirmed by a tilt table test. Participants with suspected POTS but without a diagnosis provided by a healthcare professional were excluded from this study.

### Procedure

The recruitment process involved reaching out to the mentioned Spanish patient associations through email and phone. Interested patients were notified about the study, and if they met the required criteria, they were selected for evaluation. Before participating, all the participants signed an informed consent that was sent scanned via email. After signing this document, each participant filled out a series of self-reported online surveys that collected sociodemographic and clinical data. Then, each participant engaged in an individual online assessment session in which they completed the tests while sitting and were guided by qualified neuropsychologists through the Google Meet platform, lasting around 60 min. Participants in the control group were administered the same tests as in the three clinical groups. Research was approved by the Ethics Committee of the University of Deusto (ETK-33/19-20) in 2020.

### Instruments

All neuropsychological tools included in the following assessment protocol were administered to the recruited participants. This battery of tests exhibited suitable psychometric characteristics and were adjusted for use with the Spanish population.

#### Neuropsychological assessment

**Phonetic verbal fluency test (P-M-R) and semantic verbal fluency test (“animals”)** [74]. This test is an indicator of verbal fluency. Firstly, in 60 s, the patients must pronounce words that start with the letters “P”, “M”, and “R”. Secondly, participants are asked to say as many words as they could in 60 s related to the semantic category “animals.” The calculation of the combined scores, the total number of words, from the two tests served as an overall measure of verbal fluency. The Spanish psychometric properties show Cronbach’s alpha coefficient of 0.83 [74]. A Cronbach’s alpha coefficient of 0.87 was obtained in this study.

**Stroop test** [75]. It is an indicator of processing speed and attention. In the three different parts of the test, participants are presented with a 100-word list divided into 5 columns, with 20 words in each column. The goal is to complete the tasks as quickly as possible. If the participants make a mistake, they must correct it and continue. All sections provide a total of 45 s for participants to complete the task. The number of correctly read stimuli in each section is recorded. In the first section of the test, the Stroop-W task, the word list is displayed in black ink, and assesses the reading speed of the participants. The second section, the Stroop-C subtask, assesses the participants’ visual identification of colors and processing speed. The stimuli are displayed in red, green, or blue colors. The last section, the Stroop-WC task, assesses attentional interference by measuring the speed at which individuals name colors that have incongruent color names printed on them (since it does not match the name of the written color with the color of the ink with which it is written). This part of the test requires participants to inhibit the dominant response of reading the word and instead focus on stating the color of the ink. The test–retest reliability of the Stroop test has been reported as above 0.90, 0.83, and 0.91 for each of the three parts of the test, respectively [76]. A Cronbach’s alpha coefficient of 0.87 was obtained in this study.

**Rey-Osterrieth complex figure** [77, 78]. This is a test that measures perceptual organization, visuoconstructive ability, and visual memory. The participants are asked to replicate the figure in a horizontal orientation, both by copying it and by recalling it from memory after a 3-min interval. The figure comprises 18 elements, with each element being rated from 0 to 2 on the basis of its visual precision and placement with respect to the original, resulting in a total score ranging from 0 to 36. High interrater reliability indices (between 0.80 and 0.99) are reported for the Rey-Osterrieth complex figure [79–81]. A Cronbach’s alpha coefficient of 0.69 was obtained in this study.

**The judgment of line orientation test (JLO)** [82]. This tool (Form H) assesses, by visual confrontation, the spatial relationships between line segments. The test includes 30 stimulus cards, each displaying a different pair of angled lines at the top of the page. Subjects must match these lines with those provided on a multiple-choice reference card located at the bottom of the page. The initial five items are practice items. The total number of correct responses for both line segments is recorded. Spontaneous corrections by the participant were accepted. The highest score achievable score is 30. Regarding test–retest reliability for the judgment of line orientation test, the result obtained by the authors was 0.59 [83, 84].

**The letter–number sequencing subtest of the WAIS-IV** [85, 86]. This task measures working memory. Participants are exposed to a series of numbers and letters arranged randomly. These sequences varied in length, ranging from two to eight units. Participants are instructed to recite them in a specific sequence: first the numbers in each sequence from smallest to largest, followed by the letters in alphabetical order. The addition of the correctly repeated sequences is utilized as an overall measure of working memory. The Spanish version of the subtest has presented a good internal consistency, with Cronbach’s alpha coefficient of 0.72–0.93 [85, 87]. A Cronbach’s alpha coefficient of 0.69 was obtained in this study.

**The auditory verbal learning test (WHO-UCLA AVLT)** [88, 89]. The participant’s short-term and long-term memory as well as recognition is assessed through their performance on immediate recall and delayed recall, following the learning of 15 verbal words (Trials A1–A5). They are asked to recall the words immediately (Trial A6) and again after 30 min (Trial A7). The subjects also perform a long-delayed recognition (Trial AR). The Spanish psychometric properties show Cronbach’s alpha coefficient of 0.87 [90]. A Cronbach’s alpha coefficient of 0.93 for A1–A5 and 0.89 for A6, A7 and AR was obtained in this study.

#### Psychological assessment

**Hospital anxiety and depression scale (HADS)** [91, 92]. This questionnaire assesses the presence of anxious–depressive symptomatology, commonly used in individuals with physical health conditions [91]. To this end, 14 items are asked, categorized into 2 subscales, which are scored between 0 and 3 each. The total highest score achievable is 42. The Spanish psychometric properties show Cronbach’s alpha coefficient of 0.90 for the full scale, 0.85 for the anxiety subscale, and 0.84 for the depression subscale [92]. A Cronbach’s alpha coefficient of 0.78 was obtained in this study. A recent study confirmed the reliability and validity of the anxiety and depression components of the present scale for evaluating psychological distress in individuals with LC [93].

### Data analysis

The Statistical Package for Social Sciences (SPSS) version 28.0 was used for carrying out the statistical analyses.

A Kolmogorov–Smirnov test was applied to test the normal distribution of the sample. Frequencies and descriptive statistics for both sociodemographic and clinical information were calculated. The Kruskal–Wallis test was used to compare quantitative variables between the clinical groups and the control group. In addition, the chi-squared (*χ*^*2*^) statistic was used to compare the categorical variables.

To compare the cognitive test performance and anxious–depressive symptomatology across the three clinical groups and the control group, the Kruskal–Wallis test was used together with the Dunn–Bonferroni test as post hoc analysis.

The Mann–Whitney *U* test was used to assess the potential interference of antidepressants use on cognitive outcomes.

An analysis of covariance (ANCOVA) was conducted to control for the effect of anxious–depressive symptomatology on the cognitive performance. The Bonferroni correction was applied for post hoc pairwise comparisons. For this analysis, composite scores were created by grouping indicators of anxiety and depression and converting raw scores into *Z*-values. Effect sizes were determined using the partial eta squared metric.

A significance level of *p* < 0.05 was set in all analyses.

## Results

### Sample characteristics

There were no statistically significant differences between the four groups with respect to sex [*H* (3) = 4.801, *p* =  < 0.187]; age [*χ*^2^ (3) = 3.176, *p* =  < 0.365]; nor educational level [*χ*^2^ (3) = 1.320, *p* =  < 0.724].

Regarding preexisting symptomatology, no cases of headaches or migraines were reported, nor other symptoms compatible with POTS, neither in the LC^+^ nor LC^−^ groups. Regarding prior diagnoses, three participants in the LC^+^ group had a previous diagnosis of fibromyalgia. No participants reported a prior diagnosis of POTS. In the LC^−^ group, only one participant had a previous diagnosis of fibromyalgia. None of the participants in the LC subgroups reported prior diagnoses of chronic fatigue syndrome or irritable bowel syndrome. No other prior diagnoses were reported in this group. Following COVID-19 infection, no participant in the LC clinical group was subsequently diagnosed with POTS or reported symptoms indicative of the syndrome.

The prevalence of diagnosed anxiety and depressive disorders was also examined among participants. Within the clinical LC group, eight LC^+^ and six LC^−^ participants had a formal diagnosis. These participants were the only ones taking antidepressants within their respective subgroups. Additionally, three individuals from the POTS group met the diagnostic criteria for depression, and eight met the criteria for anxiety disorders. Overall, 13 participants were taking antidepressant medication at the time of cognitive evaluation. In the healthy control group, only two participants had diagnoses of anxiety and depression, and these two were the only ones taking antidepressants.

No participant with POTS was currently undergoing any specific therapy, although some had previously received immunoglobulin treatment. In addition, the most prevalent pharmacological interventions in this clinical group were ivabradine and propranolol. Furthermore, a significant proportion of participants with POTS were advised by their healthcare providers to supplement with magnesium, folic acid, melatonin, iron, and a variety of vitamins as adjunctive therapy.

Following the description of the sample characteristics, a Kolmogorov–Smirnov (K–S) test was conducted to assess the normal distribution for the cognitive and mood variables. The majority of variables did not have a normal distribution due to the significant coefficient (K–S) value (*p* > 0.05).

The scores obtained by the three clinical groups and the control group in the neuropsychological tests are presented in Table 2. Significant differences were found between the three clinical groups and the healthy control group (*p* < 0.05). The most significant differences were found on the following cognitive scores: W, C, and WC from the Stroop Test; letters “P,” “M,” and “R” from the verbal fluency test; and Span from the letter–number sequencing subtest (*p* < 0.001). To identify which groups differed, a Dunn–Bonferroni post hoc test was conducted, which revealed significant differences on the cognitive variables between the three clinical groups and the control group (LC^+^/control, LC^−^/control and POTS/control).

**Table 2:** Clinical and control group performance on cognitive assessment and post hoc results (Dunn–Bonferroni test)

	LC^+^ group	LC^−^ group	POTS group	Control group	*H*	Dunn–Bonferroni post hoc analysis					
	*M* (SD)	*M* (SD)	*M* (SD)	*M* (SD)							
	*(n* = 30)	*(n* = 29)	*(n* = 31)	*(n* = 39)		LC^+^/LC^−^	LC^+^/POTS	LC^+^/C	LC^−^/POTS	LC^−^/C	POTS/C
WHO-UCLA-AVLT											
A1	6.63 (2.37)	5.69 (2.06)	7.03 (1.94)	7.72 (2.10)	0.001***	NS	NS	NS	NS	0.001	NS
A5	11.40 (2.92)	10.14 (3.13)	11.29 (2.71)	12.51 (2.16)	0.013*	NS	NS	NS	NS	0.007	NS
B	5.17 (1.91)	5.31 (1.80)	5.26 (2.10)	6.64 (2.17)	0.014*	NS	NS	0.04	NS	NS	NS
A6	9.90 (3.56)	8.83 (3.61)	10.42 (3.30)	11.03 (3.07)	0.059	NS	NS	NS	NS	NS	NS
A7	10.00 (3.23)	8.66 (3.67)	10.39 (3.29)	11.51 (2.71)	0.009**	NS	NS	NS	NS	0.005	NS
AR	27.73 (2.57)	26.38 (4.11)	27.10 (3.62)	28.87 (1.15)	0.016*	NS	NS	NS	NS	0.01	NS
JLO	22.17 (4.82)	23.28 (5.18)	22.06 (5.91)	25.59 (2.95)	0.015*	NS	NS	0.023	NS	NS	NS
Stroop test											
W	93.20 (22.99)	76.48 (22.38)	85.52 (23.64)	115.23 (18.42)	< 0.001***	NS	NS	0.002	NS	0	0
C	66.67 (14.58)	57.34 (18.21)	62.55 (15.62)	84.21 (13.98)	< 0.001***	NS	NS	0	NS	0	0
WC	42.87 (11.68)	37.21 (15.47)	39.39 (13.56)	58.31 (15.20)	< 0.001***	NS	NS	0.001	NS	0	0
Interference	4.47 (8.47)	4.95 (11.15)	2.95 (10.07)	12.94 (12.72)	0.003**	NS	NS	0.017	NS	NS	0.008
Verbal fluency test											
P	16.47 (5.16)	14.93 (5.57)	14.94 (4.14)	20.51 (4.02)	< 0.001***	NS	NS	0.015	NS	0	0
M	13.77 (3.99)	13.21 (4.80)	12.58 (4.24)	17.72 (4.08)	< 0.001***	NS	NS	0.005	NS	0	0
R	13.93 (4.32)	12.90 (5.33)	13.00 (4.95)	16.67 (3.59)	< 0.001***	NS	NS	NS	NS	0.001	0.002
Animals	21.27 (5.53)	19.28 (6.94)	21.77 (5.99)	24.82 (5.15)	0.003**	NS	NS	NS	NS	0.002	NS
Letter–number sequencing (WAIS-IV)											
Span	5.47 (1.31)	5.03 (1.30)	4.87 (1.31)	6.05 (0.99)	< 0.001***	NS	NS	NS	NS	0.006	0.001
Rey-Osterrieth complex figure											
Copy	31.21 (3.74)	29.93 (6.97)	28.42 (3.97)	31.68 (3.16)	0.008**	NS	NS	NS	NS	NS	0.009
Memory (immediate recall)	17.28 (5.78)	18.32 (8.17)	14.95 (6.46)	19.15 (4.35)	0.014*	NS	NS	NS	NS	NS	0.015

*A1–A5* learning trials of the original list*, A6* immediate recall of the original list*, A7* delayed recall trial of the original list*, AR* delayed recognition*, B* immediate recall of the distractor list*, H* Kruskal–Wallis test, *C* color subtest, *JLO* the judgment of line orientation test, *LC*^+^  long-COVID with a positive test group, *LC*^*−*^ long-COVID without a positive test group, *W* word subtest, *WC* word–color subtest, *WHO-UCLA-AVLT* World Health Organization-UCLA auditory verbal learning test

*p ≤ 0.05; **p ≤ 0.01; ***p ≤ 0.001

Given the use of antidepressants among the participants in this study, appropriate analyses were conducted using the Mann–Whitney *U* test to assess the potential interference of these medications on the observed cognitive outcomes. The results of these analyses did not identify any relationship between antidepressant use and the performance observed among participants in any of the cognitive tests applied (*p* > 0.05).

### Differences in anxious–depressive symptomatology between groups

Similar to the cognitive results, analysis of anxious–depressive symptomatology also revealed significant differences between the LC^+^ group (*M* = 8.52, SD = 3.56 on anxious symptomatology; *M* = 7.45, SD = 3.90 on depressive symptomatology), LC^−^ group (*M* = 11.34, SD = 3.36 on anxious symptomatology; *M* = 8.69, SD = 3.48 on depressive symptomatology), POTS group (*M* = 9.86, SD = 4.37 on anxious symptomatology; *M* = 7.93, SD = 4.59 on depressive symptomatology) and the control group (*M* = 6.54, SD = 3.26 on anxious symptomatology; *M* = 3.85, SD = 2.01 on depressive symptomatology) [*H* (3) = 25.728, *p* =  < 0.001 for anxious symptomatology; *H* (3) = 36.066, *p* =  < 0.001 for depressive symptomatology].

A Dunn–Bonferroni post hoc test identified differences between specific pairs of clinical and control groups. On the one hand, regarding anxious symptomatology, the differences were found between the pairs of LC^+^/LC^−^ (*p* adj. = 0.046), POTS/control (*p* adj. = 0.007), and LC^−^/control (*p* adj. < 0.001). On the other hand, concerning depressive symptomatology, differences were found between the pairs of LC^+^/control (*p* adj. < 0.001), POTS/control (*p* adj. < 0.001), and LC^−^/control (*p* adj. < 0.001).

### Effect of anxious–depressive symptomatology on cognitive performance

Since anxious–depressive symptomatology has the potential to impact cognitive performance outcomes, its influence was accounted for by conducting a covariance analysis (ANCOVA). The analysis was conducted across all the cognitive domains evaluated. On the basis of the results, after accounting and eliminating the effect of anxious and depressive symptomatology on cognitive assessment performance, some of the initial differences between the groups were eliminated. Firstly, the differences in the following verbal memory scores were eliminated: A5 (*p* > 0.114 controlling the effect of depressive symptomatology), B (*p* > 0.091 controlling the effect of depressive symptomatology), A6 (*p* > 0.087 controlling the effect of anxious symptomatology; *p* > 0.204 controlling the effect of depressive symptomatology), and A7 (*p* > 0.053 controlling the effect of depressive symptomatology). Secondly, differences in copy accuracy (*p* > 0.137 controlling the effect of anxious symptomatology; *p* > 0.173 controlling the effect of depressive symptomatology) and visual memory (immediate recall) (*p* > 0.055 controlling the effect of anxious symptomatology; *p* > 0.113 controlling the effect of depressive symptomatology) were also eliminated. Furthermore, after controlling for these potential confounding variables, significant differences were still found between the three clinical groups and the control group in the other cognitive variables originally considered (*p* < 0.05).

As significant results were obtained on the ANCOVA analysis, the pairwise comparisons were performed by using the post hoc analysis applying the Bonferroni correction. The results are presented in Table 3. At the post hoc analysis, new differences were identified between the clinical groups in addition to those already found in previous analyses. On the one hand, the LC^−^ group showed differences compared with the LC^+^ group in one of the Stroop test scores (*p* = 0.045) (specifically, the score obtained in W) when considering both anxious and depressive symptomatology. On the other hand, the LC^−^ also differed from the POTS group in one of the verbal memory scores (A1) (*p* = 0.041), but only when depressive symptoms were taken into consideration. Lastly, new differences were observed between the POTS group and the control group on verbal memory (*p* = 0.046) and visuospatial (*p* = 0.041 and *p* = 0.030) scores when considering both anxiety and depression symptoms into account.

**Table 3:** Bonferroni post hoc correction for the ANCOVA results

	Bonferronni post hoc analysis for anxious symptomatology						Bonferroni post hoc for depressive symptomatology					
	LC^+^/LC^−^	LC^+^/POTS	LC^+^/C	LC^−^/POTS	LC^−^/C	POTS/C	LC^+^/LC^−^	LC^+^/POTS	LC^+^/C	LC^−^/POTS	LC^−^/C	POTS/C
WHO-UCLA-AVLT												
A1	NS	NS	NS	0.041	0.004	NS	NS	NS	NS	NS	0.016	0.046
A5	NS	NS	NS	NS	0.031	NS	NS	NS	NS	NS	NS	NS
B	NS	NS	0.041	NS	NS	NS	NS	NS	NS	NS	NS	NS
A6	NS	NS	NS	NS	NS	NS	NS	NS	NS	NS	NS	NS
A7	NS	NS	NS	NS	0.015	NS	NS	NS	NS	NS	0.046	NS
AR	NS	NS	NS	NS	0.021	NS	NS	NS	NS	NS	0.029	NS
JLO	NS	NS	0.008	NS	NS	0.03	NS	NS	0.013	NS	NS	0.041
Stroop test												
W	0.045	NS	< 0.001	NS	< 0.001	< 0.001	0.045	NS	0.003	NS	< 0.001	< 0.001
C	NS	NS	< 0.001	NS	< 0.001	< 0.001	NS	NS	< 0.001	NS	< 0.001	< 0.001
WC	NS	NS	< 0.001	NS	< 0.001	< 0.001	NS	NS	< 0.001	NS	< 0.001	< 0.001
Interference	NS	NS	0.006	NS	0.02	0.002	NS	NS	0.011	NS	0.036	0.004
Verbal fluency test												
P	NS	NS	0.002	NS	< 0.001	< 0.001	NS	NS	0.01	NS	< 0.001	< 0.001
M	NS	NS	0.003	NS	0.003	0.001	NS	NS	0.008	NS	0.004	< 0.001
R	NS	NS	NS	NS	0.027	0.019	NS	NS	NS	NS	0.017	0.016
Animals	NS	NS	NS	NS	0.002	NS	NS	NS	NS	NS	0.002	NS
Letter–number sequencing (WAIS-IV)												
Span	NS	NS	NS	NS	0.013	0.003	NS	NS	NS	NS	0.021	0.007
Rey-Osterrieth complex figure												
Copy accuracy	NS	NS	NS	NS	NS	NS	NS	NS	NS	NS	NS	NS
Memory (immediate recall)	NS	NS	NS	NS	NS	NS	NS	NS	NS	NS	NS	NS

*A1–A5* learning trials of the original list, *A6* immediate recall of the original list, *A7* delayed recall trial of the original list, *AR* delayed recognition, *B* immediate recall of the distractor list, *C* color subtest, *JLO* the judgment of line orientation test, *LC*^+^ long-COVID with a positive test group, *LC*^*−*^ long-COVID without a positive test group, *W* word subtest, *WC* word–color subtest, *WHO-UCLA-AVLT* World Health Organization-UCLA auditory verbal learning test

## Discussion

Years after the onset of the COVID-19 pandemic, several studies have focused on studying the persistent effects of this infection on cognition. However, it has been difficult to reach a clear conclusion, due to counterproductive results in the different studies already published. In addition, there is now an emerging need to better define LC and its associated complications, and to differentiate it from other post-viral syndromes [94]. Hence POTS, a syndrome most commonly triggered by viral infections, also associated with cognitive difficulties and highly prevalent among patients with LC, becomes a suitable option to achieve this objective. For all these reasons, and because these difficulties seem to disrupt the daily activities of both clinical groups [95–98], it is necessary to contribute to the identification and definition of the shared difficulties in each clinical group. Therefore, in the present study, the cognitive performance of two groups of patients with LC (with and without a positive test) and a group of patients with POTS was compared with healthy control participants, matched by age, sex, and years of education.

The results of the current study provide corroboration of difficulties in different memory typologies in patients with LC, in addition to patients with POTS. First, verbal memory impairments were identified in the three clinical groups, findings that are consistent with previously published studies with LC [99–104]. Second, the three clinical groups in this study show a worse visual memory in comparison with their healthy peers, consistent with some of the studies already published with patients with LC [102, 103, 105] and patients with POTS [106, 107]. However, not all studies have replicated these results in the LC population [108].

The three clinical groups also showed poorer visuospatial abilities (visual perception, construction, and copying accuracy) compared with their healthy peers. These results are consistent with those obtained by Díez-Cirarda et al. [65] in patients with LC. However, the findings from the current study in the POTS clinical group are in contrast to the results of Loughan et al. [109], in which the results of two patients were average.

While all these cognitive domains show deficits, the most significant differences between the three clinical groups and the control group were found in their performance on the following executive functions skills: attention, processing speed, and verbal fluency (letters P and M) (according to *p*-values, *p* < 0.001).

Regarding the attention deficits identified in the present study, already published papers on patients with LC coincide with these results [65, 100, 102, 103]. Studies conducted with patients with POTS have also identified attentional deficits in this population [33, 37, 62, 106, 109].

The mean scores obtained in the current research reflect lower inhibition control capacity both in patients with LC and with POTS compared with the control group. Although some studies have not identified inhibitory difficulties in these populations either in the LC [110] or POTS populations [109], other inquiries are in line with our outcomes [103]. However, despite the scores obtained in the present study on inhibition capacity being significantly lower in comparison with healthy control participants, they remain at the expected mean, which is consistent with what was observed in a study conducted with patients with LC [102].

Additionally, the findings from the present study indicate significant impairments in processing speed and verbal fluency. Firstly, regarding the findings on processing speed, these are consistent with studies carried out with patients with LC [100, 102] and with POTS [33, 109]. Secondly, in relation to verbal fluency, the results from the current study indicate how this domain seems to be impaired in the two clinical groups, findings that are in line with what has been observed in other studies with patients with LC [101–103, 110–113] and with POTS [109]. Nevertheless, studies such as that of Arnold et al. [33], with patients with POTS, do not observe differences on verbal fluency between these clinical groups and the groups composed of healthy control participants.

Finally, working memory deficits were detected in the current study, both in patients with LC and with POTS, similar to what has already been identified in previous literature with the LC [65, 101, 102, 112] and POTS populations [59–61, 63, 109].

Significant differences were also found between the four groups in terms of anxious–depressive symptomatology. Subsequent statistical analysis indicated that the significant group differences in anxious and depressive symptoms were primarily observed between the three clinical groups and the control group. There was only one difference between two of the clinical groups in terms of anxiety levels. Specifically, the LC^−^ group had a statistically higher mean for anxiety levels compared with the LC^+^ group.

After controlling the effect of anxious–depressive symptomatology, some of the observed differences were eliminated and other were added. On the one hand, the differences identified in verbal and visual memory were eliminated, detecting an influence of these symptoms on verbal and visual memory and copy accuracy. The results revealed that anxiety symptoms significantly influenced the cognitive performance of patients with POTS, while depressive symptoms affected the cognitive performance of all three clinical groups compared with the control group. In this way, the findings from the present research are consistent with other studies conducted with patients with LC and POTS [34, 104], in which an association between cognitive results and psychological variables has also been noted. On the other hand, extra differences were added after the post hoc analysis, namely between the LC^+^/LC^−^, POTS/LC^−^ and POTS/control comparison pairs on verbal memory, attention, and processing speed scores. The mentioned results provide further evidence for the influence of anxious–depressive symptomatology in the LC^−^ and POTS clinical groups. These results also suggest that participants of the LC^−^ group may differ, in part, from the LC^+^ subgroup. Therefore, the present study highlights the existence of intragroup variability by assessing the importance of the influence of the emotional state on the perception of symptom intensity, as the LC^−^ group (who, a priori, exhibited higher levels of worry and suggestibility), exhibits characteristics suggestive of patients with hypochondriacal traits, although further studies are needed to confirm this finding.

To date, the causes that could be generating these cognitive difficulties in both clinical groups are still unknown. However, some recently published studies have already began to consider and test hypotheses to explain these difficulties. On the one hand, regarding studies conducted with patients with LC, results from Díez-Cirarda et al. [65] and Serrano del Pueblo et al. [102] were able to identify relationships between GM atrophy and volume loss, functional connectivity alteration, and reductions of the integrity of white matter with cognitive dysfunction. These structural changes are related, in particular, to deficits of attention, verbal memory (learning and recall), working memory, processing speed, and performance on visuospatial tests [65, 103].

On the other hand, finding the cause of cognitive deficits in patients with POTS proves challenging due to a scarcity of research in this area. While a single neuroimaging study [114] identified structural changes in brain regions that have been linked to the attentional cognitive domain (putamen and cingulate gyrus) [115, 116], the link of these brain changes to cognitive performance remains unexplored in these patients. Additionally, studies suggest dysfunction in the norepinephrine system clearance present in patients with POTS [117, 118] that produces elevated circulating levels of norepinephrine (NE) [119, 120]. It is well known that NE plays a crucial role in various cognition functions, including the attentional domain [121]. Despite NE’s established role in attention, studies such as that of Arnold et al. [33] found no link between NE levels and cognitive performance in patients with POTS, suggesting that dysregulation of NE does not significantly contribute to cognitive impairment. Furthermore, results from different studies suggest a link between decreased cerebral perfusion and cognitive impairments in patients with POTS [122–124]. Thus, more research is needed to elucidate the mechanisms underlying the detected cognitive deficits in the LC and POTS clinical populations.

This is the first study to compare the cognitive profile of patients with LC and with POTS. This study addresses a gap in the literature by studying the cognitive performance in clinical populations of LC and POTS. The findings from the present study demonstrate that both clinical groups exhibit poorer cognitive performance compared with the control group. The results reveal a significant association between anxious–depressive symptomatology levels in the LC^−^ and POTS groups and specific cognitive variables such as verbal memory and attention.

Thus, the current findings, combined with previous research demonstrating cognitive impairments in these populations, suggest a promising avenue for developing innovative cognitive interventions for both LC and POTS on the basis of their specific cognitive needs. An early identification of cognitive impairments could enable timely intervention, potentially preventing or mitigating long-term consequences. Additionally, given the apparent interference of anxious–depressive symptomatology on cognitive performance in these populations, it is essential to consider concurrent psychological interventions. A comprehensive approach that addresses both cognitive and emotional aspects of these conditions may yield the most beneficial outcomes.

Certain limitations must be considered when interpreting the results of this study. First, the sample was not randomized, as the participants were recruited from different Spanish associations of affected patients. Secondly, due to pandemic conditions and concerns about the health of the clinical sample, the cognitive assessment was conducted using online methodology. Although the validity of this procedure has been demonstrated in previous studies [125, 126], it would be convenient to conduct face-to-face assessments as well. Third, the neuropsychological tests selected for the study are in most cases not the same as those used in previous studies, which makes it difficult to generalize these conclusions. Fourthly, for the POTS group, it is important to note that the cognitive tests were conducted in a seated position. This gives reason for caution in interpreting the results, as previous studies on cognitive function in this population have used different positions when performing the tasks: supine, semi-recumbent, or seated [33, 98]. Furthermore, comorbidities of the sample make it difficult to generalize these conclusions. Moreover, due to the cross-sectional design used in the study, it is not the possible to interpret causality and identify the development of these neuropsychological symptoms over the course of the diseases. This would require the establishment of a follow-up. Lastly, it is important to acknowledge the cultural differences between the patient cohort presented in this study and samples of other international populations. Thus, cross-cultural comparisons may hinder the generalizability of our findings.

For future research, it would be valuable to include a larger number of participants and investigate the effects of variables or factors such as fatigue, sleep quality, or pain level to gain a more comprehensive understanding of other aspects that could influence cognitive performance. In addition, future research could focus on including a different control group comprising individuals who have recovered from COVID-19, both with and without serological evidence of infection, to explore the potential psychological impact of the pandemic itself, beyond the direct effects of the viral infection. Furthermore, it would be interesting to use imaging techniques, together with the applied neuropsychological tests, which could provide new insights and corroborate these results.

## Conclusions

The present study is the first to compare and analyze the cognitive performance between patients with LC and POTS and healthy adults of the same age, sex, and educational level. The findings from this study show worse cognitive functioning in patients with LC and POTS in comparison with their healthy peers. Higher rates of anxious–depressive symptomatology are also found in the three clinical groups compared with the control group. The results suggest that anxious–depressive symptoms may significantly influence the cognitive performance of patients with LC and POTS. After controlling for these psychological factors, working memory, attention, and verbal fluency emerged as the most affected cognitive domains.

These cognitive deficits can significantly impair activities of daily living, affecting their social interactions, academic performance, and occupational functioning. First, difficulties with attention can lead to impaired concentration (i.e., difficulty following a conversation, the plot of a TV show, or a book) and decreased productivity. Second, working memory deficits may hamper the ability to follow complex conversations or hinder problem-solving, decision-making, and learning new skills. Third, impaired verbal fluency directly impacts communication, both written and spoken. The impact of these cognitive deficits extends beyond daily activities and can significantly compromise health management. In this way, difficulties with memory and attention can lead to missed appointments, forgotten medication schedules, and challenges in understanding and following complex treatment plans. Challenges with medication adherence, resulting from these cognitive deficits, can compromise treatment efficacy and increase the risk of adverse health outcomes.

These results highlight the importance of addressing both cognitive and psychological aspects in the management of LC and POTS. Healthcare professionals may benefit from considering a comprehensive approach that includes not only cognitive interventions, but also psychological interventions aimed at reducing anxiety and depressive symptoms in these patient groups, since on the basis of the present findings, they seem to influence their cognitive performance.

## Data Availability

The data presented in this study are not publicly available because they belong to the University of Deusto, but are available on request from the corresponding author (Aitana Ruiz de Lazcano).
